# An alveolar macrophage-targeted ciprofloxacin polymeric prodrug improves survival in a murine model of *Klebsiella pneumoniae* pneumonia

**DOI:** 10.1128/aac.00363-25

**Published:** 2025-12-16

**Authors:** Ciana L. López, Guilhem Rerolle, Sarah Snyder, Osvaldo Arias, Abdullah Bashmail, Giovany Gonzalez, Debashish Roy, Brian Lee, Jessica M. Snyder, Isabella Doorn, Sarah M. Baker, Shelton W. Wright, T. Eoin West, Shawn J. Skerrett, Patrick S. Stayton

**Affiliations:** 1 Department of Bioengineering, University of Washington 7284, Seattle, Washington, USA; 2 Division of Pulmonary, Critical Care and Sleep Medicine, Department of Medicine, University of Washington 7284, Seattle, Washington, USA; 3 Department of Comparative Medicine, University of Washington 7284, Seattle, Washington, USA; 4 Division of Allergy and Infectious Diseases, Department of Medicine, University of Washington 7284, Seattle, Washington, USA; 5 Division of Pediatric Critical Care Medicine, Department of Pediatrics, University of Washington 7284, Seattle, Washington, USA; University of Pittsburgh School of Medicine, Pittsburgh, Pennsylvania, USA

**Keywords:** *Klebsiella pneumoniae*, alveolar macrophages, polymeric prodrugs, pneumonia, nanomaterials

## Abstract

*Klebsiella pneumoniae* is a leading cause of both hospital- and community-acquired pneumonia. Nanomaterials have the potential to deliver antibiotics directly to sites of infection with improved pharmacokinetics and to avoid development of antimicrobial resistance. We previously demonstrated the use of alveolar macrophage (AM)-targeted polymeric prodrugs to prevent pneumonia in murine models caused by the facultative intracellular pathogens *Francisella novicida* and *Burkholderia pseudomallei*. These fully synthetic mannose-tagged polymers engage with AM mannose receptors, permitting uptake and triggering intracellular ciprofloxacin release. Here we show that the AMs can also serve as a reservoir for releasing antibiotics to treat infections caused by a primarily extracellular bacterium. Aerosolized ciprofloxacin polymeric prodrugs significantly improved survival in a murine model of *K. pneumoniae* pneumonia, reduced lung bacterial burden, lessened extent of lung injury, and prevented excessive neutrophilic inflammation.

## INTRODUCTION

Pneumonia is a leading cause of morbidity and mortality worldwide and is the primary cause of death by infectious agents in children under 5 years of age (1). In 2019 alone, three primarily extracellular pulmonary pathogens (*Streptococcus pneumoniae*, *Staphylococcus aureus*, and *Klebsiella pneumoniae*) resulted in more than 1.5 million deaths globally (2). Compounded by the SARS-CoV-2 pandemic, the social and economic impact of pneumonia is high (3).

*K. pneumoniae*, a Gram-negative opportunistic pathogen, is an important cause of hospital-acquired pneumonia (4). Its propensity for conjugative transfer of virulence factors such as multiple drug resistance (MDR) and hypervirulence has warranted its categorization by the World Health Organization as a critical, priority 1 threat (5). In its classical form, *K. pneumoniae* infects patients with comorbidities including alcohol use disorder and diabetes mellitus. In its hypervirulent form, the bacteria cause severe community-acquired infections in healthy individuals worldwide (6). Unfortunately, mortality rates are greater than 40% for resistant strains (7).

Treatment involves oral or intravenous antibiotics and is associated with prolonged hospitalization and high costs (8). High systemic antibiotic dosing is necessary to overcome suboptimal lung biodistribution and poor pharmacokinetics (9, 10). For example, ciprofloxacin—a commonly used oral fluoroquinolone antibiotic—has a plasma elimination half-life of 4 h. Moreover, systemic antibiotics can cause serious adverse side effects including neurotoxicity, ototoxicity, nephrotoxicity, and tendon rupture (11). Given the stagnation in antibiotic development in the past 50 years, there is a critical need to improve the delivery of existing antibiotics to maximize antimicrobial efficacy and minimize the development of resistance.

Direct pulmonary drug delivery is a convenient treatment alternative to localize antibiotics to the site of bacterial persistence, avoid systemic toxicities, and prevent first-pass metabolism (12). Inhalable antibiotics have been approved by the U.S. Food and Drug Administration (FDA) to deliver agents effective against *Pseudomonas aeruginosa* directly to the lungs and treat infections associated with cystic fibrosis (CF). However, despite dramatically improved drug localization via direct delivery, rapid clearance from infected lung tissues still results in suboptimal pharmacokinetic profiles and requires frequent dosing regimens (13).

Aerosolized nanomaterial formulations, such as liposomes, have been developed with the goal of extending antibiotic half-life in the lung and enhancing intracellular uptake (14–16). Others demonstrated that ciprofloxacin could be formulated into nanocrystals and encapsulated in aerosolizable liposomes to extend drug release to allow for less frequent administrations (17). Liposomal formulation of ciprofloxacin enhanced phagocytosis and improved treatment in murine models of *Yersinia pestis*, which, dependent on the stage of infection, moves from intracellular niches to the extracellular space as disease progresses (15). The clinical benefit of aerosolized nanomaterials was solidified in 2018 when the FDA approved an inhalable liposomal amikacin (aminoglycoside antibiotic) suspension to treat *Mycobacterium avium* complex lung disease. Since then, inhalable liposomal ciprofloxacin formulations have received FDA orphan drug designation for treatment of bronchiectasis and CF (18). Despite these important successes, widespread adoption of liposomal drug formulations has been slow due to complicated synthetic schemes (19, 20) and low shelf stability (21).

Polymeric prodrugs are an attractive alternative for local, controlled antibiotic delivery (22–24). Our group has shown that attaching drugs to polymerizable monomers enables their combination with other functional monomers in precise ratios, thereby improving targeting, solubility, and controlled drug release (25–28). These features result in enhanced pharmacokinetic properties and increased treatment effectiveness within a single macromolecule. These polymeric prodrugs use one-pot synthetic schemes that result in low product dispersity (29) and high reproducibility (30) compared with other nanomaterials that require multi-step conjugation schemes to achieve multifunctionality (31–34). In the context of lung infections, inhalable ciprofloxacin-containing polymeric prodrugs provided total protection in lethal murine models of pulmonary tularemia and melioidosis caused by facultative intracellular pathogens *Francisella novicida* and *Burkholderia pseudomallei* (35–37). Intracellular bacteria residing in alveolar macrophages (AMs) were targeted using multivalent hydrophilic mannose comonomers with high affinity for the CD206 receptor. Once the polymeric prodrugs were endocytosed by AMs, a protease-cleavable valine-citrulline (VC) linker facilitated the intracellular release of ciprofloxacin and effective clearance of AM bacterial reservoirs. Pharmacokinetic analysis showed that aerosolized AM-targeted polymeric prodrug treatment resulted in a 6.5-fold higher maximum ciprofloxacin concentration within AMs compared to the aerosolized free drug (36). After a single dose of the prodrug, ciprofloxacin remained detectable in AMs for up to 1 week, whereas the free drug treatment showed no detectable ciprofloxacin in most mice at all time points (37). Notably, the polymeric prodrug also improved ciprofloxacin pharmacokinetics beyond the intracellular compartment, producing an 8.4-fold increase in the area under the concentration-time curve (AUC_0-24 h_) in the whole lung compared to the free drug, which was eliminated from the lungs by 2 h (36). This is likely attributed to the membrane permeability of ciprofloxacin, and these findings indicate that such macrophage-targeted polymeric prodrugs may also be well suited for extracellular infections like *K. pneumoniae*.

**Fig 1:**
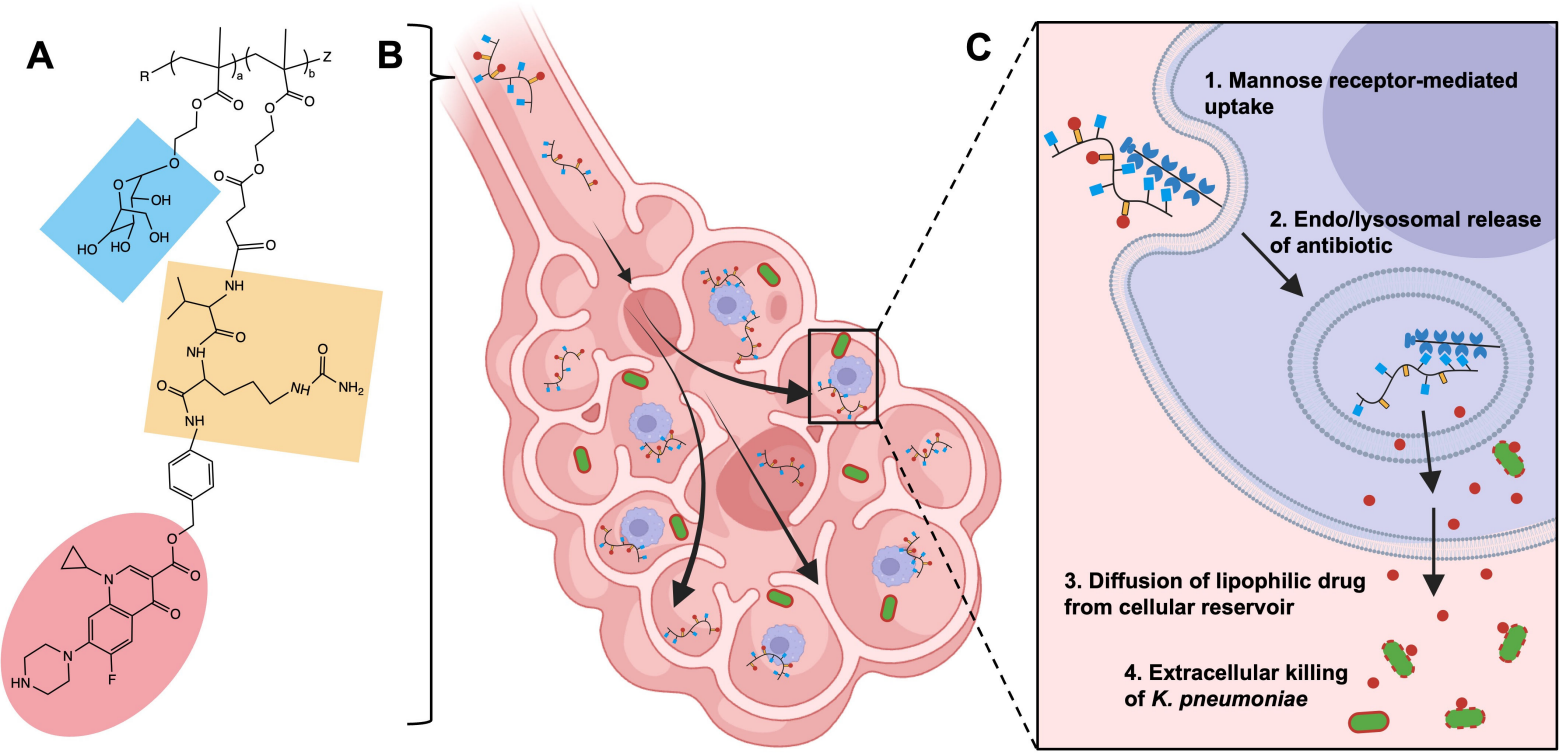
AM-targeted polymeric prodrugs can be used to treat extracellular pulmonary infections like *K. pneumoniae*. The schematic describes (**A**) a fully synthetic polymeric prodrug that incorporates functional monomers that target AMs via mannose (blue) and intracellular protease cleavage domains (yellow) that trigger the release of antibiotic cargo (ciprofloxacin, red). (**B**) The ciprofloxacin polymeric prodrug is administered directly to the lungs as an aerosol, and (**C**) upon mannose-receptor (CD206)-mediated uptake by AMs, the antibiotic is released intracellularly to form cellular drug reservoirs from which the lipophilic drug can diffuse locally to eliminate extracellular *K. pneumoniae* infection. Figure developed using BioRender.com.

Here, we propose the therapeutic use of an aerosolized ciprofloxacin polymeric prodrug for the treatment of pulmonary infections caused by *K. pneumoniae*. We hypothesize that by targeting ciprofloxacin release within local AM reservoirs and the diffusion of lipophilic antibiotic locally, the improved ciprofloxacin pharmacokinetics within the lung will extend survival (Fig. 1). Traditionally considered a primarily extracellular pathogen, growing evidence suggests that facultative intracellular survival of *K. pneumoniae* within AMs may play a significant role as a virulence mechanism, further validating our rationale for an AM-targeted approach (38–40). Local application of targeted polymeric prodrugs to lethal and resistant infections like *K. pneumoniae* could provide a solution for patients with severe disease.  

## RESULTS

The ciprofloxacin polymeric prodrug was synthesized by reversible addition fragmentation chain-transfer (RAFT) co-polymerization of mannose-MA and VC-linked Cipro-MA (VC-cipro) as previously described (36, 37). The purified polymer was characterized by ^1^H NMR spectroscopy. The peaks corresponding to mannose and VC-cipro were observed (Fig. S1A). Monomer feed ratios are shown in Table S1. ^1^H NMR analysis indicated 86% monomer conversion, and analysis of the purified polymer with a levofloxacin standard indicated a ciprofloxacin drug weight of 9% (Fig. S1B). The number average molecular weight, M_n_, was determined by size exclusion chromatography to be 24.2 kDa. The ciprofloxacin polymeric prodrug had a narrow molecular weight distribution with dispersity, Đ = 1.1 (Fig. S1C).

**Fig 2:**
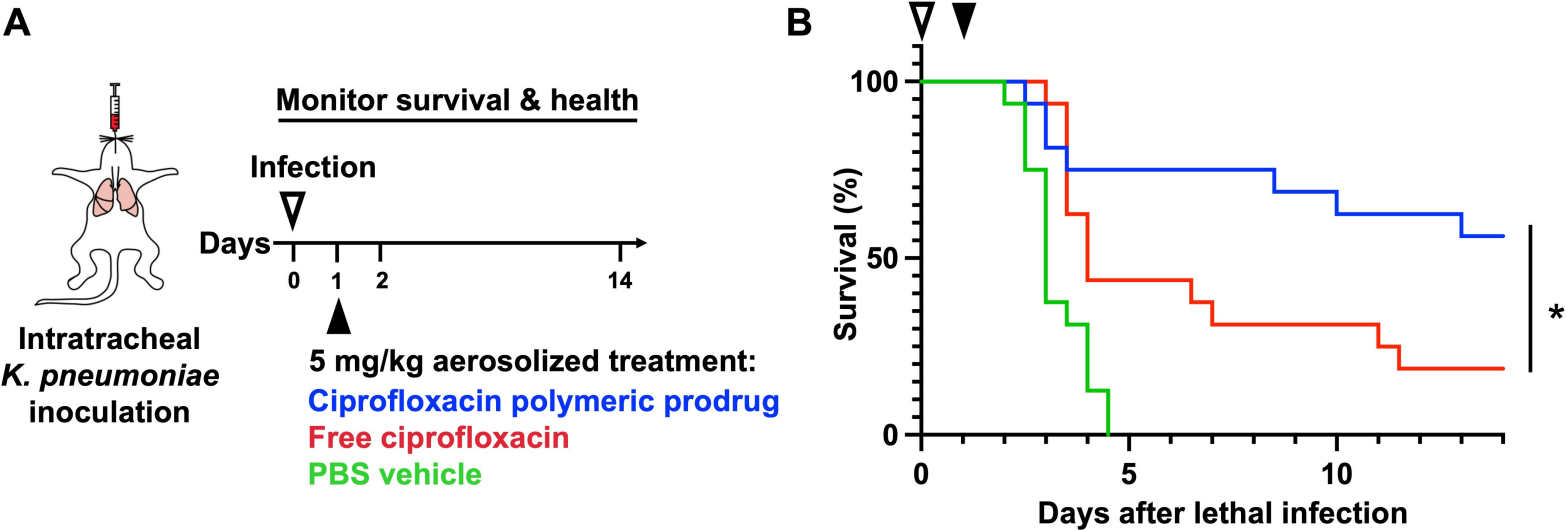
Therapeutic treatment with the ciprofloxacin polymeric prodrug significantly improves mouse survival against a lethal *K. pneumoniae* infection. (**A**) *In vivo* model workflow schematic. Twenty-four hours after *K. pneumoniae* infection (5–9 × 10^3^ CFU), free ciprofloxacin, the ciprofloxacin polymeric prodrug, or PBS vehicle was administered as a single dose to albino C57BL/6 mice via intratracheal aerosolization using a MicroSprayer (*n* = 16 per treatment group). (**B**) Survival comparing mice receiving the ciprofloxacin polymeric prodrug to those treated with free ciprofloxacin was monitored for 14 days, where * indicates a statistically significant improvement in survival using ciprofloxacin polymeric prodrug compared to free ciprofloxacin treatment (*P*-value = 0.04, as assessed by log-rank test). The results are the combination of two separate experiments with *n* = 8 per group each.

Preliminary experiments showed that increasing the frequency of aerosolized free ciprofloxacin treatment (5 mg/kg dose) in a murine model of lethal *K. pneumoniae* pneumonia led to improved survival (Fig. S2), thereby suggesting that a local and sustained polymeric prodrug formulation would have improved efficacy. The therapeutic activity of the macrophage-targeted ciprofloxacin polymeric prodrug was investigated in a murine model of *K. pneumoniae* pneumonia. Mice were inoculated intratracheally (IT) with a lethal dose of *K. pneumoniae* (5–9 × 10^3^ CFU), and infection was established for 24 h. By this point, *K. pneumoniae* bacterial burden in the lungs is well-established, with bacterial loads on the order of 10^6^ CFU per lung (Fig. S3). In most mice, bacteria were also detected beyond the lungs, as indicated by elevated bacterial loads in the spleen and liver. These results support the clinical relevance of the model since clinical infections often disseminate rapidly beyond the pulmonary space in a similar manner (41). At 24 h post-infection, mice were treated with the ciprofloxacin polymeric prodrug (56 mg/kg, equivalent to 5 mg/kg ciprofloxacin, *n* = 16) IT using a MicroSprayer (Fig. 2A). Control mice received either free ciprofloxacin or phosphate buffered saline (PBS) vehicle treatment (*n* = 16 per group). The polymer demonstrated exceptionally improved solubility compared to the free ciprofloxacin, which required additional formulation with 5% dextrose in water. The survival data indicated a significant survival benefit associated with the ciprofloxacin polymeric prodrug treatment compared to the free ciprofloxacin (Fig. 2B). As expected, no mice in the PBS control group survived to the 14-day end point, with 100% of mice (16/16) meeting the euthanasia criteria by day 5. Most mice that received the free ciprofloxacin treatment also met the euthanasia criteria by day 5 (56%), and only 3/16 mice survived until the end of study. In comparison, 75% of the ciprofloxacin polymeric prodrug-treated mice survived past 5 days (12/16) and 9/16 mice (56%) survived to the end of the experiment. To evaluate the impact on survival of the polymer backbone and mannose targeting moieties, a control polymer containing only mannose monomer and none of the ciprofloxacin prodrug monomer was synthesized with a similar molecular weight (16.3 kDa) (^1^H-NMR analysis shown in Fig. S1D). The mannose control polymer showed no significant survival benefit or detriment in the lethal *K. pneumoniae* infection model (Fig. S4) with a similar dose of mannose to the ciprofloxacin polymeric prodrug used in previous studies, supporting our hypothesis that the survival benefit associated with the ciprofloxacin polymeric prodrug is the result of the targeted and sustained release of ciprofloxacin within the lungs.

**Fig 3:**
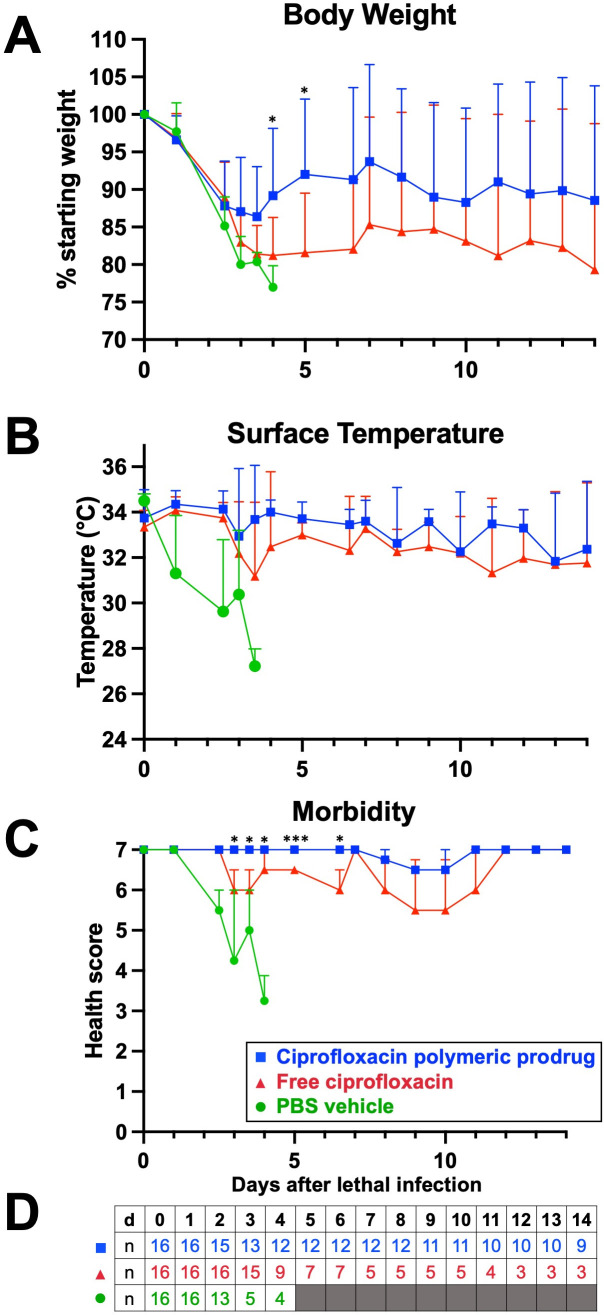
Health conditions of *K. pneumoniae*-infected mice (5–9 × 10^3^ CFU) after treatment at 24 h post-infection with the ciprofloxacin polymeric prodrug (5 mg/kg ciprofloxacin equivalent), free ciprofloxacin (5 mg/kg), or the PBS vehicle. The body weight (**A**), surface temperature (**B**), and clinical scores (**C**) of the mice were monitored over the course of the 14-day experiment. The clinical scores of mice are based on activity, coat, breathing, posture, eyes, isolation, and resistance to handling. (**D**) All treatment groups started with *n* = 16 mice, and the number of mice per group (n) by the end of the day (d) is indicated. % starting weight and temperature data are presented as mean and standard deviation; comparison between the ciprofloxacin polymeric prodrug and free ciprofloxacin groups at indicated time points was assessed by unpaired t test. Health score data are presented as median and interquartile range; comparison between the ciprofloxacin polymeric prodrug and free ciprofloxacin groups at indicated time points was assessed by Mann-Whitney test, where significance is indicated by *P* < 0.05 (*), and *P* < 0.001 (***). The results are the combination of two separate experiments with *n* = 8 per group each.

Parameters including changes to body weight, ventral surface temperature, and overall clinical score (36) based on isolation, handling, posture, coat, eyes, breathing, and activity were measured over the course of survival studies. The weight was the most consistent indicator of morbidity, and the weight of mice from all treatment groups declined by >10% within the first 3 days, indicating that all mice developed an infection (Fig. 3A). The mice treated with the ciprofloxacin polymeric prodrug regained weight with significant improvements compared to mice treated with the free ciprofloxacin and PBS on days 4 and 5 post-infection. Surface temperature was a less predictive, but more imminent, indicator of mortality, as evidenced by the rapid reduction in PBS-treated mice (Fig. 3B). Treatment with the free ciprofloxacin was also correlated with lower surface temperatures compared to the ciprofloxacin polymeric prodrug. The clinical scores of infection (Fig. 3C) indicated a significant improvement in morbidity in mice treated with the ciprofloxacin polymeric prodrug beginning on day 3 post-infection compared to those treated with free ciprofloxacin and up until day 6.5. Most surviving mice treated with the ciprofloxacin polymeric prodrug maintained scores of between 6 and 7 throughout the study (maximum/healthy score = 7) compared to mice treated with free ciprofloxacin, which trended lower (between 5 and 6 for surviving mice). Mice receiving PBS control rapidly displayed clinical signs of morbidity by day 3 (score ≤ 4 met criteria for euthanasia by this metric). For clarity, a table is included with the number of mice in each group surviving at each time point since more mice succumbed to infection at later time points (Fig. 3D).

**Fig 4:**
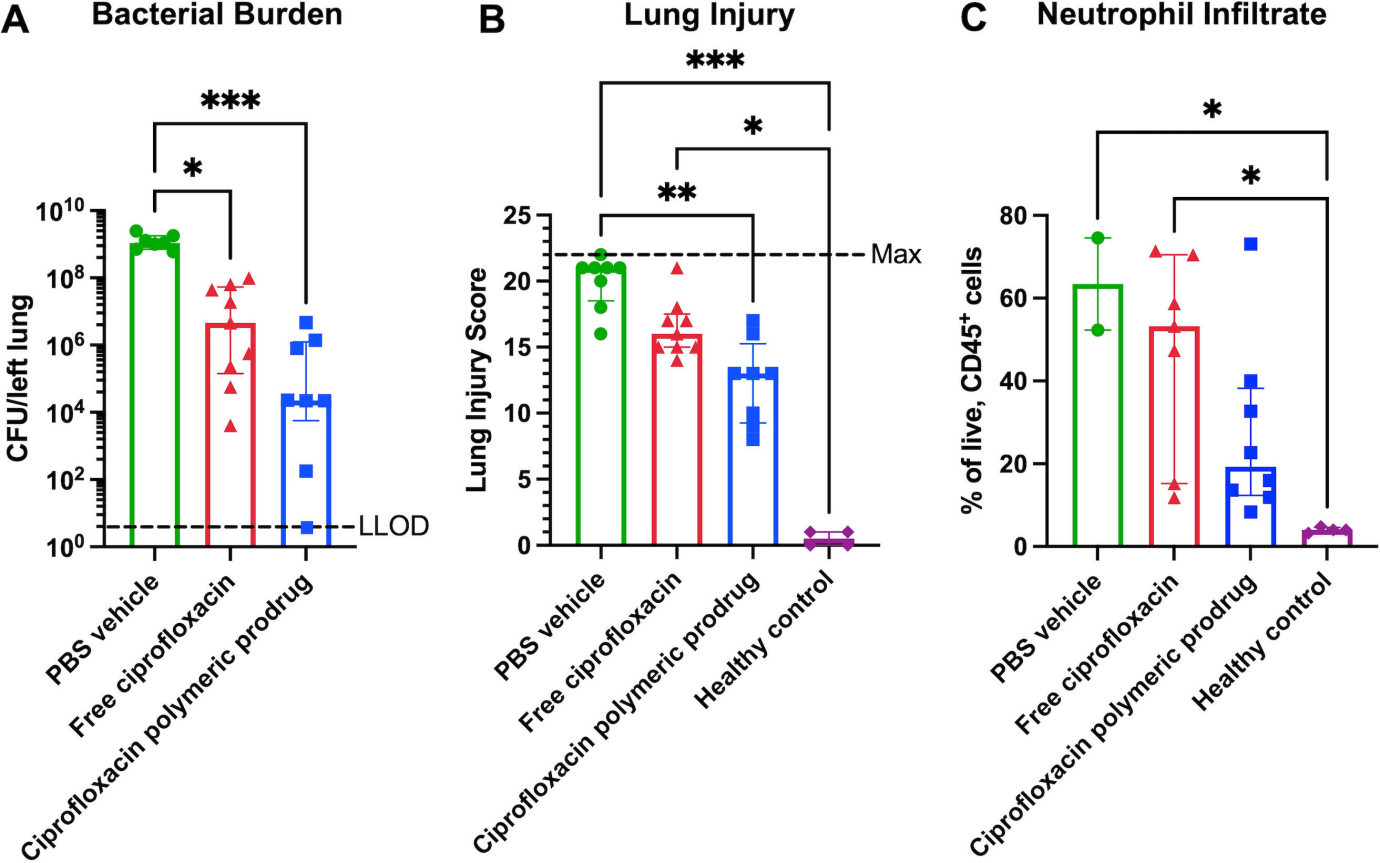
Bacterial burden, extent of lung injury, and amount of neutrophilic inflammation are reduced in *K. pneumoniae*-infected mice treated with the ciprofloxacin polymeric prodrug 24 h after infection. Lung bacterial burden (**A**) and lung injury scores (**B**) were evaluated 72 h after infection with 6–7 × 10^3^ CFU *K*. *pneumoniae*/48 h after treatment with the ciprofloxacin polymeric prodrug, free ciprofloxacin, or the PBS control (*n* = 7–9 per treatment group, *n* = 4 for healthy controls). The lower limit of detection (LLOD) was 10 CFU per left lung. Lung injury scores were based on histopathological scoring (Table S2) on the extent of inflammation, edema, bacteria visualized, and overall involvement of the lung and compared to four uninfected healthy controls (maximum score = 22). Four days after the *K. pneumoniae* infection (650–750 CFU) and 3 days after the ciprofloxacin polymer prodrug, free ciprofloxacin, or PBS treatment (*n* = 2–6), neutrophil infiltrate populations in lung tissues (**C**) were compared. The LLOD = <1% based on FMO gating. Bars and error bars represent the median and interquartile range, respectively. Statistical significance was assessed by the Kruskal-Wallis test with Dunn’s multiple comparisons, where significance is indicated by *P* < 0.05 (*), *P* <0.01 (**), and *P* < 0.001 (***).

The bacterial burdens in the lung were compared at 72 h post-infection (equivalent to 48 h post-treatment) to determine the improved antimicrobial effect of the ciprofloxacin polymeric prodrug (Fig. 4A). The mice treated with the ciprofloxacin polymeric prodrug had two orders of magnitude reduction in lung bacterial burden (median = 2.3 × 10^4^ CFU/left lung) compared to mice treated with the free ciprofloxacin (median = 4.6 × 10^6^ CFU/left lung). The ciprofloxacin polymeric prodrug treatment led to a five orders of magnitude and a statistically significant (*P* = 0.002) improvement compared to PBS vehicle-treated mice (median = 1.1 × 10^9^ CFU/left lung). Treatment with free ciprofloxacin also resulted in significantly reduced bacterial burden compared to PBS vehicle treatment but with lower statistical significance (*P* = 0.0182) than treatment with the ciprofloxacin polymeric prodrug.

Histopathological analysis was conducted on the right lungs at 72 h post-infection to assess the extent of the lung injury associated with each treatment and compared to healthy controls. The lung injury scoring criteria are described in Table S2. Mice that received the ciprofloxacin polymeric prodrug suffered significantly less lung injury (*P* = 0.0049) compared to mice treated with PBS control (Fig. 4B), whereas there was no significant difference between mice treated with PBS or free ciprofloxacin. Furthermore, while mice treated with PBS control and free ciprofloxacin showed significantly increased lung injury scores compared to healthy controls (*P* = 0.0002 and *P* = 0.0293, respectively), there was no significant difference in the lung injury scores between mice treated with ciprofloxacin polymeric prodrug and healthy controls. Mice that received the ciprofloxacin polymeric prodrug had less overall lung involvement (Fig. S6) compared to mice treated with the free ciprofloxacin, largely attributed to significantly reduced inflammatory cell infiltration in the bronchiolar lumen and similar trends in the alveolar and interstitial spaces. The scores for each specific criterion are shown in Fig. S6.

Neutrophilic inflammation can eliminate *K. pneumoniae* through several mechanisms; however, *K. pneumoniae* is able to impede efferocytic uptake and removal of neutrophils by macrophages, which can result in persistent inflammation (42). Thus, we sought to determine the impact of the ciprofloxacin polymeric prodrug on pro-inflammatory cytokine levels in the lungs. Cytokine measurements at early time points (24, 48, and 72 h) indicated similar reduction trends of tumor necrosis factor-alpha and interleukin-17 (IL-17A) production in mice treated with the free ciprofloxacin and ciprofloxacin polymeric prodrug compared to the PBS controls (Fig. S7A and B). IL-17A levels in mice treated with free ciprofloxacin were significantly reduced at 48 h compared to the PBS control (*P* = 0.0023) but were not significantly different by 72 h. The production of C-X-C motif chemokine ligand 2 (CXCL2), a neutrophil chemoattractant, in mice treated with the ciprofloxacin polymeric prodrug and free ciprofloxacin was significantly different compared to treatment with PBS controls at 72 h post-infection/48 h post-treatment (*P* = 0.0003, *P* = 0.0004, respectively) (Fig. S7C). Further, a trending decrease in CXCL2 over time in mice treated with the ciprofloxacin polymeric prodrug deviated from mice treated with free ciprofloxacin and PBS controls, which showed increasing CXCL2 levels over time, suggesting that the improved and sustained polymeric drug delivery compared to free ciprofloxacin could be minimizing the extent of neutrophilic inflammation associated with infection.

To determine if there was reduced inflammation and restored homeostasis in mice at later time points following treatment with the polymeric prodrug compared to free ciprofloxacin, neutrophil populations were quantified at 4 days post-infection and subsequent treatments compared to healthy controls. In this study, a lower inoculum (650–750 CFU) was required in order for enough mice per group to survive to 4 days post-infection. Mice treated with the ciprofloxacin polymeric prodrug 24 h after infection had a median neutrophil infiltrate of 19% of live, CD45+ cells, which was not significantly different from healthy controls (4%); in contrast, mice treated with free ciprofloxacin showed significantly increased neutrophil infiltration (53%, *P*-value = 0.0165), similar to PBS vehicle controls (63%, *P*-value = 0.0235) (Fig. 4C). Macrophage populations comprised a smaller fraction of the CD45+ cells and did not significantly differ between the ciprofloxacin polymeric prodrug and free ciprofloxacin treatment groups. However, there was a significant difference between the PBS vehicle and uninfected healthy controls (*P*-value = 0.0415), indicating that the assay captured the dynamic range of macrophage infiltration (Fig. S8B). Furthermore, regardless of the treatment group, individual mice with increased bacterial burdens had macrophage and neutrophil populations that generally trended lower and higher, respectively (Fig. S8C and D).

**Fig 5:**
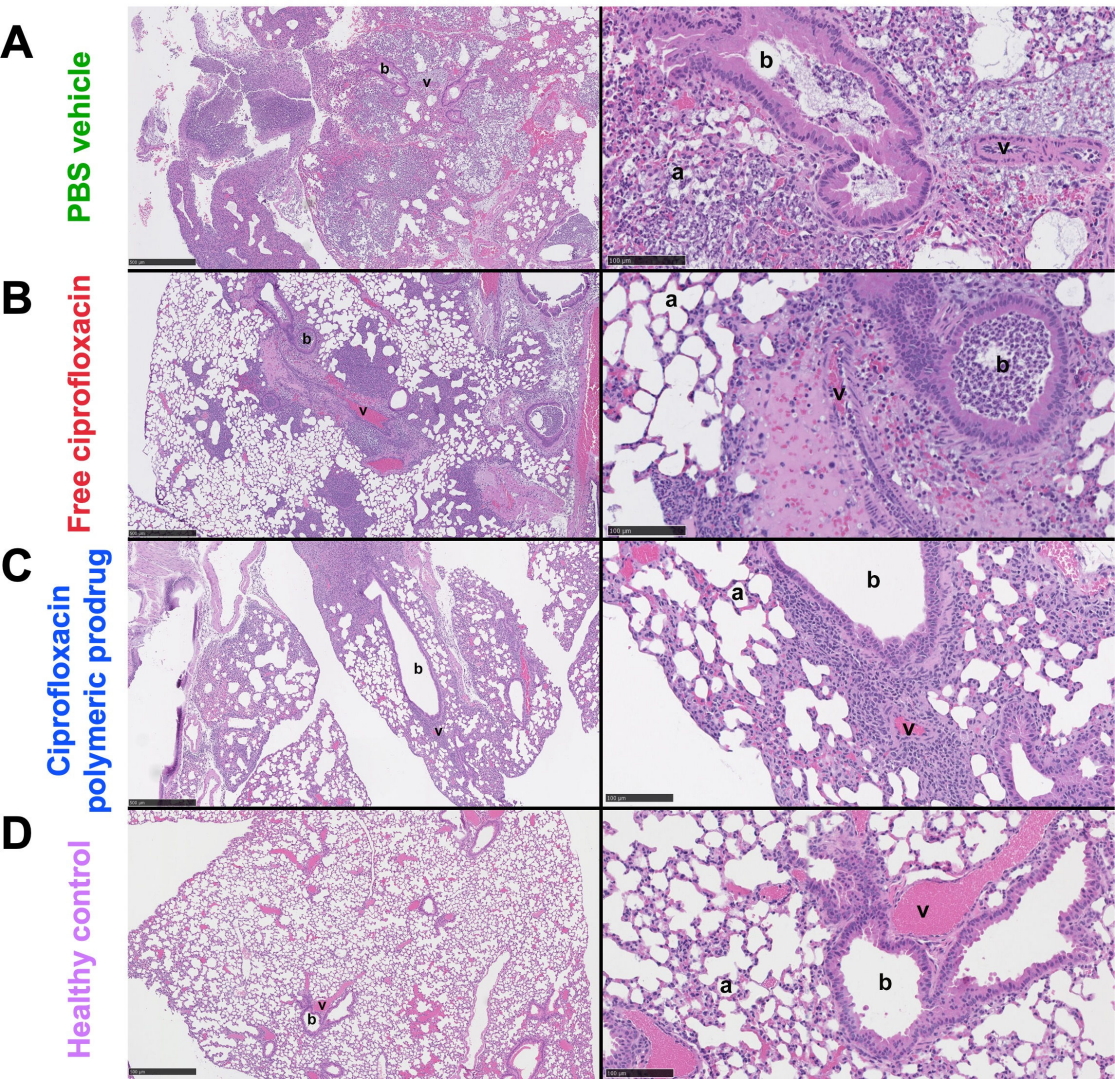
Histological images of *K. pneumoniae*-infected mice harvested at 72 h post-infection (6–7 × 10^3^ CFU) and following treatment at 24 h post-infection with (**A**) PBS vehicle, (**B**) free ciprofloxacin, or (**C**) the ciprofloxacin polymeric prodrug. (**D**) Uninfected control mice. From left to right scale bar size = 500 µm, 100 µm. a, alveolar space; b, bronchiolar lumen; v, vessel lumen.

The capability of ciprofloxacin polymeric prodrug treatment to decrease pathology associated with *K. pneumoniae* was evident from the histopathological analysis (Fig. 5). Improvements were observed in the alveolar space in mice treated with the ciprofloxacin polymeric prodrug (Fig. 5C, left and right panels) compared to treatment with PBS and free ciprofloxacin (Fig. 5A and B, left and right panels) with fewer inflammatory cells within alveoli and reduced disruption and loss of underlying alveolar wall structure associated with necrosis and hemorrhage. Abundant bacteria were visualized in the PBS vehicle-treated lung and to a lesser degree in the free ciprofloxacin-treated mice (Fig. 5A and B; Fig. S5). A notable difference in treatment between the ciprofloxacin polymeric prodrug and free ciprofloxacin was also seen in the bronchiolar lumen, which was indicated by a significantly reduced percentage of airway impacted by inflammatory cell infiltrate (right panels of Fig. 5B and C; Fig. S6), indicating that polymeric prodrug treatment helps prevent bronchiolar inflammation.

## DISCUSSION

Overall, this study demonstrates that incorporating antibiotic into a targeted polymeric prodrug formulation can improve treatment of a primarily extracellular bacterial infection such as *K. pneumoniae*. In a murine model of pulmonary infection, treatment with the ciprofloxacin polymeric prodrug extended survival, reduced bacterial burden and extent of lung injury, and restored lung homeostasis. We propose that this is due to the improved pharmacokinetic properties of the polymeric prodrug formulation afforded by both CD206 targeting and intracellular drug cleavage (36, 37) with no designed mechanism for extracellular release other than the innate lipophilicity of the antibiotic. The use of the mannose-tagged polymeric prodrug, delivered IT, shifts the intrapulmonary distribution of antibiotic cargo toward resident AMs. The findings here suggest that sufficient antibiotic is released to improve survival against a primarily extracellular *K. pneumoniae* infection. While synthetic polymeric prodrugs are tested here in a *K. pneumoniae* model, the results suggest the promise of similar polymer formulations against other primarily extracellular infections that have been identified as the biggest contributors to global mortality (e.g., *S. pneumoniae*, *S. aureus*) and at reasonable synthetic costs compared to other antibiotic targeting mechanisms (2, 43, 44).

While these results are promising, there are some important limitations to discuss. First, we used a single, standard, susceptible bacterial strain that has been extensively studied in pneumonia models (45–50). Future studies should examine multiple *K. pneumoniae* strains (50, 51). Second, because of the lethality of this infection model, many readouts were restricted to surviving animals, which introduces selection bias. Future studies using sublethal murine infection models could allow for more gradual disease progression and extended observation (52). Third, we did not include a control treatment group that received systemic therapy as our focus was inhalation therapy (15, 53–56).

This study used an intratracheal MicroSprayer to administer aerosolized treatment in our murine model of pneumonia. In a nosocomial setting, direct IT administration of polymeric prodrug might be feasible, but inhaler or nebulizer formulations should also be evaluated. This may open opportunities for effective and accessible outpatient care. Additionally, while a single aerosolized polymeric prodrug treatment improved survival for mice with *K. pneumoniae* pneumonia here, it is conceivable that multiple doses would further improve survival benefits.

More effective treatments for pneumonia are urgently needed, especially with the continued emergence of antimicrobial resistance. While ciprofloxacin is the small molecule drug incorporated here, the inhalable polymeric prodrug platform could provide an avenue for local delivery of other antibiotics with limited clinical utility due to high systemic toxicity (e.g., polymyxins and aminoglycosides) despite high potency against MDR *K. pneumoniae* infections. Further, while an antibiotic polymer was evaluated herein, the polymer prodrug platform offers opportunities to combine antimicrobial and host-directed agents. These targeted polymeric prodrugs may have broad applicability for lower respiratory tract infections by combining local drug delivery with optimized pharmacokinetics and wielding the potential to reduce antibiotic-associated resistance and toxicity.

## MATERIALS AND METHODS

### RAFT synthesis of ciprofloxacin polymeric prodrug and mannose control polymer

The co-polymerization of poly(mannose-co-VC-cipro) was conducted from methacrylate monomers as previously described (36, 37). The polymerization reaction was conducted under a nitrogen atmosphere in dimethyl sulfoxide (DMSO) at 70°C using the chain transfer agent (CTA), 4‐cyano‐4‐[(ethylsulfanylthiocarbonyl)sulfanyl]pentanoic acid (ECT), and the radical initiator (I) 4,4′‐azobis(4‐cyanovaleric acid). The initial monomer (M) to CTA ratio ([M]_0_/[CTA]_0_) was 40:1, and the initial CTA to initiator ratio ([CTA]_0_/[I]_0_) was 10:1. The molar ratio of mannose to VC-Cipro-MA monomer was 85:15 (Table S1). The reaction was run for 4 h followed by precipitation in diethyl ether and dialysis against phosphate buffer and then distilled water (6 days total; 3,500 Da molecular weight cutoff (MWCO)). The polymer was lyophilized and passed through a PD-10 desalting column before being lyophilized into a final, purified solid product that was confirmed by ^1^H NMR spectroscopy and size exclusion chromatography (Fig. S1A and C). Drug weight determination by ^1^H NMR is described in supporting information methods. A mannose methacrylate control polymer (lacking the drug-containing VC-cipro-MA comonomer) was synthesized via RAFT polymerization was conducted in DMSO-d6. An initial [M]_0_/[CTA]_0_ was 56:1 with a [CTA]_0_/[I]_0_ of 1:0.16. Mannose-MA (520 mg, 1.779 mmol) and ECT (8.37 mg, 0.0318 mmol) were added in a 5 mL round bottom flask and dissolved in DMSO-d6 (total 1,040 µL). V70 (1.57 mg, 0.005 mmol) was added from a stock solution. After purging under nitrogen for 40 min, the flask was placed in a pre-heated oil bath at 42°C for 23 h. The overall monomer conversion was obtained at approximately 99%. The crude polymer was purified via precipitating in diethyl ether/acetone (70/30 vol/vol) mixture and then dialyzed against water (24 h) using a 3.5-kDa MWCO regenerated cellulose membrane. The resulting solution was flash frozen and then lyophilized. The polymer was further purified via PD10 desalting column and lyophilized to obtain powder polymer before being lyophilized into a final, purified solid product that was confirmed by ^1^H NMR spectroscopy (Fig. S1D).

### Animals

Female B6(Cg)-Tyrc-2J/J mice, 8–12 weeks old, were purchased from The Jackson Laboratory and maintained at the University of Washington under specific pathogen-free conditions. Mice were acclimatized for at least week prior to experimentation and provided *ad libitum* access to food and water.

### Bacterial growth conditions and quantification of bacterial burden

*K. pneumoniae* strain 43816 was purchased from The American Type Culture Collection. Bacteria were plated on tryptic soy (TS) agar, and a single mucoid colony was isolated. Stocks were prepared in 20% glycerol in 1× PBS and stored at −80°C until use. For mouse experiments an overnight culture of 0.25 mL *K*. *pneumoniae* in 25 mL TS broth was incubated in a 125-mL flask shaking at 200 rpm at 37°C with aeration and grown for 18 h to stationary phase. Bacteria were harvested, washed three times in PBS (4°C), and diluted to a final concentration of 500–6000 CFU *K*. *pneumoniae* in a 50-µL inoculation volume based on spectrophotometer measurements (OD_600_ = 0.3 was determined to be equivalent to 3.3 × 10^8^ CFU/mL). Gram stains were used to confirm the purity of the sample. Bacterial cultures were quantified by plating serial dilutions and incubated at 37°C for 18 h after which colonies were counted. The bacterial burdens in the lungs (left lung), spleen, and liver (median hepatic lobe) were quantified at 24 h post-infection. The lung bacterial burden was also quantified at 72 h post-infection. For mice that did not survive to the designated time points and were euthanized upon meeting criteria, organs were harvested and plated to assess bacterial burden. Organs were harvested in Omni tubes containing 1 mL PBS and homogenized for 35 s using a Bead Ruptor homogenizer (Omni, Inc). Serial dilutions were plated on TS agar in duplicate, and the number of colony-forming units was quantified after 18-h incubation at 37°C. The CFU/lung, CFU/spleen, and CFU/liver were calculated based on the weight of the harvested organ.

### *K. pneumoniae* pulmonary infection model

Mice were inoculated IT with a target dose of 6,500 CFU *K*. *pneumoniae* (procedure described in supporting information). Doses were validated by plating serial dilutions of inoculum; a table reporting the target and experimental inoculum associated with each study is included in Table S3. Twenty-four hours later, mice were treated with 5 mg/kg free ciprofloxacin, the ciprofloxacin polymeric prodrug equivalent (9 wt% cipro, 56 mg/kg polymer), or the PBS vehicle control (*n* = 16 per treatment group). The ciprofloxacin HCl (Alfa Aesar) was formulated in a 5% D-glucose (Sigma) in molecular grade water (Corning) solution. The ciprofloxacin polymeric prodrug was formulated in PBS (Sigma). For the mannose control polymer study, the polymer was formulated in PBS (22 mg/kg to match the dose of mannose associated with ciprofloxacin polymeric prodrug from previous studies) and compared against the PBS vehicle control (*n* = 8 per treatment group). All treatment solutions were filter sterilized (0.22 µm, Millex C#SVG012SL, Millipore Sigma) and administered in a 50-µL total volume using a MicroSprayer aerosolizer (Model IA-1C, Penn-Century, Inc) to mice under 4% isoflurane anesthesia. Mice were monitored at least 1–2 times daily based on predetermined euthanasia criteria, including (1) ventral surface temperature below 24°C, (2) weight loss exceeding 25%, and (3) an overall activity score ≤4 based on a 7-point scale assessing isolation, handling, posture, coat, eyes, breathing, and activity; upon reaching criteria in two out of these three metrics or at defined endpoints prior to this point, mice were euthanized with pentobarbital (300 mg/kg intraperitoneally), followed by exsanguination by cardiac puncture.

### Histopathological analysis

Right lungs of surviving mice were collected 72 h after infection and submerged in 10% formalin (Sigma) at a volumetric ratio of 1:10 that was refreshed at 24 h and replaced with 70% EtOH at 48 h. Samples were paraffin embedded, sectioned at 4 µm, and stained with hematoxylin and eosin by the University of Washington Histology and Imaging Core. Lung lesions were semi-quantitatively scored in a blinded fashion by a board-certified veterinary pathologist based on the degree of perivascular inflammation and edema on a scale of 0–4, bronchiolar lumen (0–4), alveolar (0–4) and interstitial (0–4) inflammation, extent of bacteria visualized (0–2, as shown in Fig. S5), and extent of disease in the lung (0–4), for a maximum total score of 22. Full scoring parameters are listed in Table S2.

### Statistical analysis

Depending on whether the data were parametric or nonparametric, results are reported as mean and standard deviation or as median and interquartile range, respectively, and were analyzed using the appropriate statistical methods. A *P*-value < 0.05 was considered statistically significant, and the significance was reported as follows: *P* < 0.05 (*), *P* < 0.01 (**), and *P* < 0.001 (***). All analyses were performed using GraphPad Prism, v9. Investigators were not blinded to group allocation except for histologic lung injury scoring, which was blinded.

## SUPPLEMENTAL MATERIAL

Supplemental Material aac.00363-25-s0001.docx Supplemental methods, Fig. S1 to S8, and Tables S1 to S3.

ASM does not own the copyrights to Supplemental Material that may be linked to, or accessed through, an article. The authors have granted ASM a non-exclusive, world-wide license to publish the Supplemental Material files. Please contact the corresponding author directly for reuse.
