# Supplementary material for: An alveolar macrophage-targeted ciprofloxacin polymeric prodrug improves survival in a murine model of Klebsiella pneumoniae pneumonia
Source: Antimicrob Agents Chemother. 2025 Dec 16;70(2):e00363-25. doi: 10.1128/aac.00363-25 (PMC12888862; doi:10.1128/aac.00363-25)
Supplement: Supplemental Material — Supplemental methods, Fig. S1 to S8, and Tables S1 to S3. [file aac.00363-25-s0001.docx]

**Supporting Information**

An alveolar macrophage-targeted ciprofloxacin polymeric prodrug improves survival in a murine model of *Klebsiella pneumoniae* pneumonia

Ciana L. López,**^1^** Guilhem Rerolle,^2^ Sarah Snyder,^1^ Osvaldo Arias,^1^ Abdullah Bashmail,^2^ Giovany Gonzalez,^2^ Debashish Roy,^1^ Brian Lee,^2^ Jessica M. Snyder,^3^ Isabella Doorn,^2^ Sarah M. Baker,^4^ Shelton W. Wright,^5^ T. Eoin West,^2^ Shawn J. Skerrett,^2^ and Patrick S. Stayton^1#^

^1^Department of Bioengineering, University of Washington, Seattle, WA

^2^Division of Pulmonary and Critical Care Medicine, Harborview Medical Center, University of Washington, Seattle, WA

^3^Department of Comparative Medicine, University of Washington, Seattle, WA

^4^Division of Allergy and Infectious Diseases, Department of Medicine, University of Washington, Seattle, WA

^5^Division of Pediatric Critical Care Medicine, Department of Pediatrics, University of Washington, Seattle, WA

^#^Address correspondence to Patrick Stayton, stayton@uw.edu.

**Supplemental Methods**

*^1^H NMR determination of polymer drug weight percentage:* The ciprofloxacin drug weight percentage was analyzed by spectroscopy (Bruker AV 300) in deuterated dimethyl sulfoxide (DMSO-d6) using an internal standard, levofloxacin. Briefly, 7.2 mg polymeric prodrug was dissolved in levofloxacin solution (DMSO-d6, 1 mg/mL, 0.7 mL) and analyzed using ^1^H NMR spectroscopy. Molar composition of ciprofloxacin polymeric prodrug was determined by comparing a single proton resonance from the internal standard at δ = 8.9 ppm against a single proton resonance from ciprofloxacin polymeric prodrug at δ = 8.42 ppm (Figure S1 B).

*Size exclusion chromatography (SEC):* SEC was used to determine molecular weight and dispersities (M_w_/M_n_, Đ) of the ciprofloxacin polymeric prodrug. The purified polymer was dissolved at 5 mg/mL in the running buffer (0.15 M sodium acetate buffered to pH 4.4 with acetic acid) for analysis by SEC. Samples were then applied to an OHpak SB-804 HQ column (Shodex) in line with a miniDAWN TREOS light scattering detector (Wyatt) and an OptiLab rEX refractive index detector (Wyatt). Absolute molecular weight average (M_n_) was calculated based on dn/dc value calculated separately for the polymer using ASTRA software (Wyatt).

*Intratracheal inoculation of K. pneumoniae:* Mice were anesthetized with 5% isoflurane (1 L/min O_2_) for 4 minutes and then positioned on an incline board, suspended by incisors. Vocal cords were visualized by laryngoscope and with an external illuminator. Mice were intubated with a 22 g catheter attached to a manometer (tuberculin syringe prepared with 100 µL of PBS) to confirm airway access. The manometer was carefully removed and replaced with a tuberculin syringe pre-loaded with 50 µL stationary-phase bacterial suspension in PBS and 150 µL air, which was slowly depressed to complete instillation. Mice were monitored for anesthesia recovery.

*Cytokine measurements of left lung*: Mice were infected with 6 x 10^3^ CFU *K. pneumoniae* and treated 24 hours later with 5 mg/kg free ciprofloxacin, the ciprofloxacin polymer prodrug (9 wt% ciprofloxacin, 56 mg/kg), or PBS control. Left lungs were harvested at 24 hours (pre-treatment), 48 hours, and 72 hours (n = 3 – 4 per timepoint). Left lung homogenates were lysed in a 1:1 lysis buffer solution consisting of 15mM Tris-HCl, 1.5mM EDTA, 150mM NaCl, 1% Triton, and protease inhibitors (Complete Protease Inhibitor cocktail; Roche) on ice for 30 minutes, centrifuged at 1500 g for 15 minutes at 4°C. Supernatants were collected and stored at -80°C until analysis using the following R&D DuoSet^®^ ELISA kits: Mouse CXCL2 (Catalog# DY452-05), Mouse IL-17A DuoSet ELISA Kit (Catalog# DY421-05), Mouse TNF (Catalog# DY410-05), and Mouse IFN-γ (Catalog# DY485-05).

*Analysis of neutrophil populations:* Mice were inoculated with 650-750 CFU *K. pneumoniae* and treated 24 hours later with 5 mg/kg free ciprofloxacin, ciprofloxacin polymeric prodrug equivalent (9 wt% ciprofloxacin, 56 mg/kg) or PBS vehicle control (n = 8 per treatment group). Daily health monitoring was conducted, mice were euthanized as they met criteria. At the 4-day endpoint remaining mice were euthanized, the left lung was collected to determine bacterial burden. The right lung was perfused with 5mL of PBS through the right ventricle. Harvested lung tissue was minced and digested as previously described.^27, 28^ Briefly, lungs were treated with 200K/mL DNase I (Roche) and 1 mg/mL liberase TM (Roche) in Roswell Park Memorial Institute (RPMI, Sigma) media at 37°C while shaking at 250 rpm for 45 minutes. Lung digest was gently mashed through a 70 µm cell strainer (Fisher) and washed with RPMI and PBS. Cells were pelleted by centrifugation, red blood cells (RBCs) were lysed (RBC lysis buffer, eBio), and remaining cells processed for flow cytometry according to the gating scheme in Figure S8A. “Fluorescence minus one” controls were used to determine gating scheme as shown in Figure S8 and adapted from previous publications.^1, 2^ In brief, doublets, debris, and dead cells (stained with Live/Dead Aqua BV510, Invitrogen) were excluded from analysis and immune cells were identified using the pan-hematopoietic marker, CD45 (stained with CD45 APC-Cy7 clone 30-F11, Biolegend). Of CD45+ cells, Neutrophils were identified as Ly6G+, CD11b+ (stained with Biolegend Ly6G FITC clone 1A8 and CD11b PE-Cy7 clone M1/70). Of non-neutrophils, alveolar macrophages were identified as CD11c+ (stained with CD11c PE clone N418, Biolegend) and SiglecF+ (stained with Siglec-F PerCP eFluoro-710 clone 1RNM44N, eBioscience).

**Supplemental Figures**

**Figure S1**. Ciprofloxacin polymeric prodrug synthesis. (A) Representative ^1^H-NMR (300 MHz, dimethyl sulfonoxide-d_6_ (DMSO-d6)) of synthesized ciprofloxacin polymeric prodrug with Val-Cit dipeptide linker, poly(Man-co-VC-cipro). (B) Representative ^1^H-NMR (300 MHz, DMSO-d6) of synthesized ciprofloxacin polymeric prodrug with Val-Cit dipeptide linker, poly(Man-*co*-VC-cipro) with an internal standard, Levofloxacin, to determine the drug weight percentage. (C) Gel permeation chromatography of ciprofloxacin polymeric prodrug using differential refractive index detector. (D) Representative ^1^H-NMR of synthesized mannose control polymer, poly(mannose), in DMSO-d6.

**Table S1.** Monomer feed ratios of poly(Man-co-VC-cipro), referred to as ciprofloxacin polymeric prodrug, and mannose control polymer. DP = degree of polymerization.

**Figure S2.** Repeated doses of free ciprofloxacin improve survival against lethal *K. pneumoniae* infection indicating that a targeted polymeric prodrug with sustained release might improve therapeutic efficacy. A) *In vivo* model workflow schematic. Free ciprofloxacin (5 mg/kg) or D5W were intratracheally administered (50 𝜇L aerosolization) to albino C57BL/6 mice at 2, 24, and 48 hours after intratracheal infection (6 x 10^3^ CFU *K. pneumoniae*) using a MicroSprayer® (n = 5 per treatment group). Survival (B) was monitored for 14 days. 5% dextrose in water (D5W).


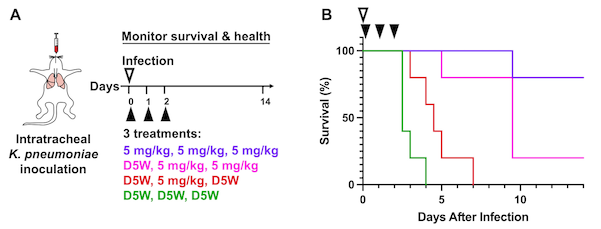


**Figure S3.** Bacterial dissemination in the left lung, spleen, and liver at 24 hours post intratracheal infection (6 x 10^3^ CFU *K. pneumoniae*). The bars and error bars represent the median and interquartile range of n = 4 mice, respectively. The lower limit of detection (LLOD) was 10 CFU per organ.


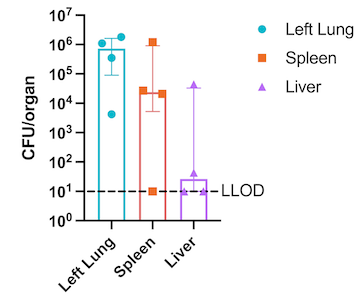


**Figure S4.** Mannose control polymer impact on mouse survival against lethal *K. pneumoniae* infection. A) In vivo model workflow schematic. 1X PBS vehicle or mannose control polymer (22 mg/kg) were intratracheally administered (50 𝜇L aerosolization) to albino C57BL/6 mice at 24 hours after intratracheal infection (5 x 10^3^ CFU *K. pneumoniae*) using a MicroSprayer® (n = 8 per treatment group). B) Survival was monitored for 4.5 days until all mice met the criteria for euthanasia. No significant difference was found as assessed by log-rank test. The body weight (C), surface temperature (D) and clinical scores (E) of the mice were monitored over the course of the experiment. % starting weight and temperature results are presented as mean and standard deviation and health score results are presented as median and interquartile range.

**
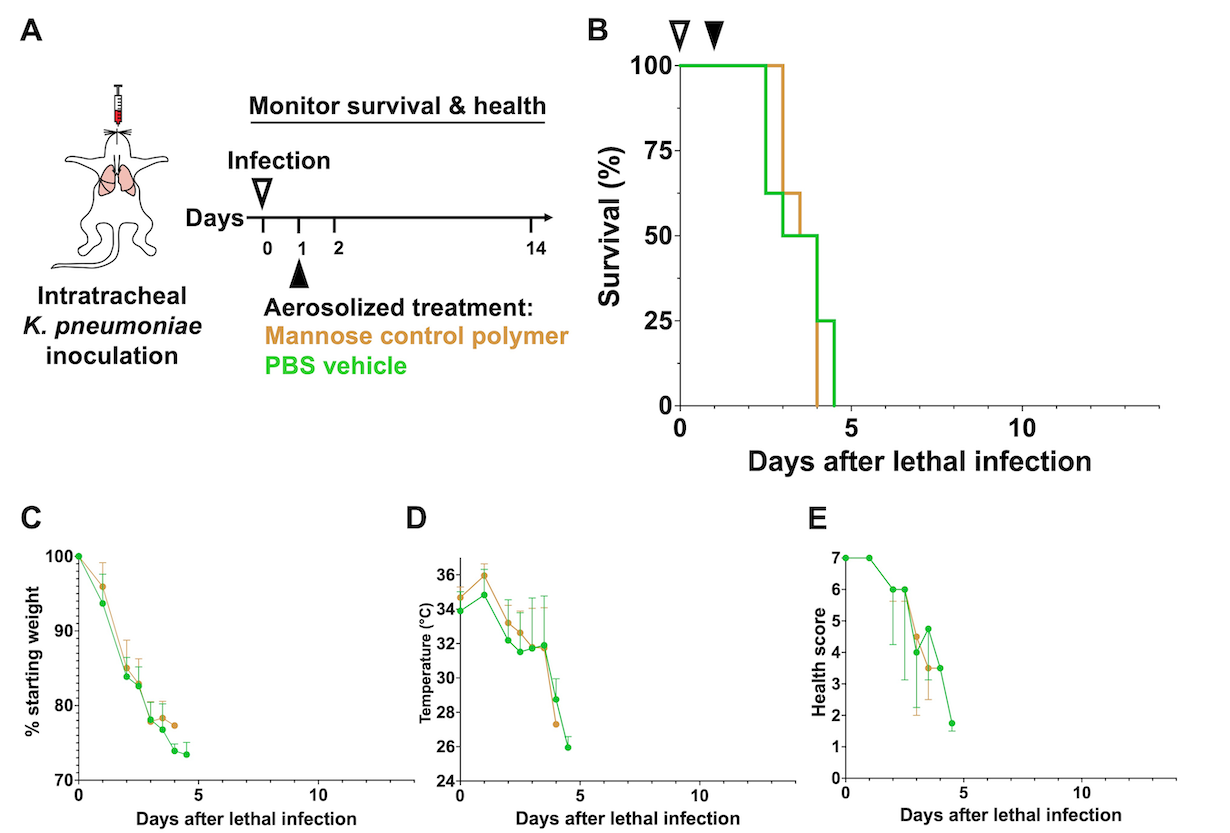
**

**Table S2.** Histologic lung injury scoring criteria.

**Figure S5.** Annotated visualization of bacteria within alveolar airway at 72 hours post-infection and 48 hours post-treatment. Images at 600X magnification show representative sample tissues from A) healthy mice with score 0, B) mouse treated with the ciprofloxacin polymeric prodrug with score 0, C) mouse treated with free ciprofloxacin with score 1, and D) mouse treated with PBS vehicle with score 2. Scale bar = 10 µm.

**
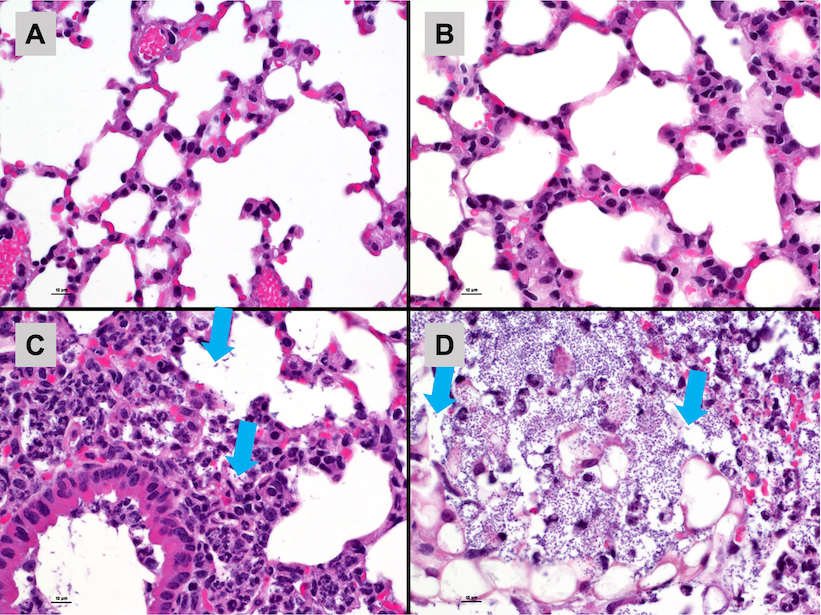
**

**Figure S6.** Lung injury scoring breakdown for mice 72 hours after lethal *K. pneumoniae* infection (6 – 7 x 10^3^ CFU) and treatment at 24 hours post-infection with the ciprofloxacin polymeric prodrug, free ciprofloxacin, or PBS vehicle and compared to healthy control. The line and error bars represent the median and interquartile range, respectively. Statistical significance was assessed by Kruskal-Wallis test with Dunn’s multiple comparisons.

**
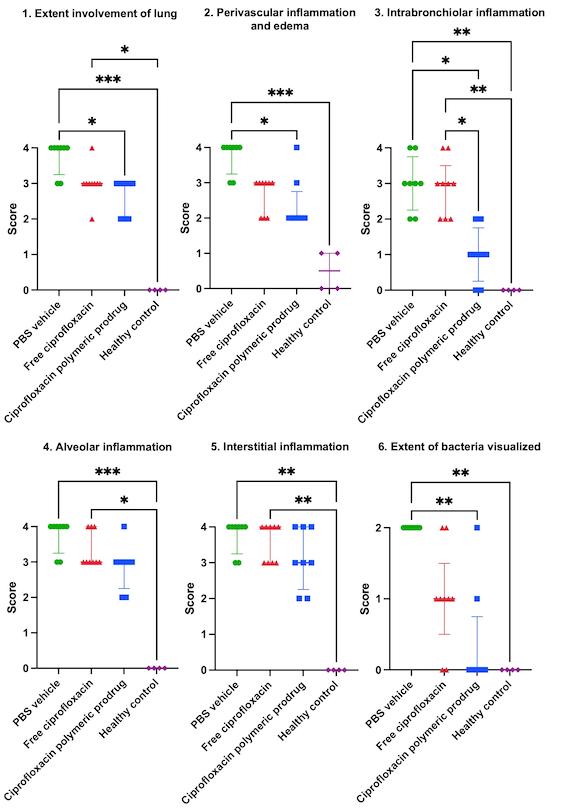
**

**Figure S7.** Cytokine levels of (A) tumor necrosis factor-alpha (TNF), (B) IL-17A, and (C) CXCL2 were measured by ELISA at 24, 48, and 72 hours after intratracheal infection of albino C57BL/6 mice with 6500 CFU *K. pneumoniae* and microsprayer treatment at 24 hours with 5 mg/kg ciprofloxacin as free drug or from polymeric prodrug and compared to PBS vehicle control. Data presented as median and interquartile range (n = 3 – 4). Interferon gamma (IFN-γ) was also measured but were below the detection limit of the assay (31.2 pg/mL). The line and error bars represent the mean and standard deviation, respectively. Statistical analysis was determined by a mixed effects model.

**
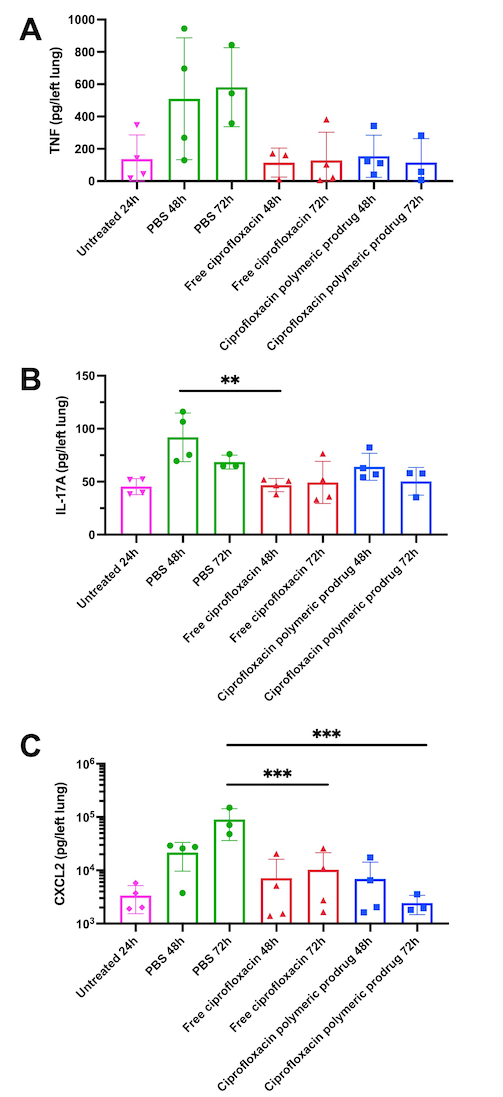
**

**Figure S8.** (A) Gating scheme for flow cytometry analysis of right lungs harvested from *K. pneumoniae*-infected mice at four days post infection (650 – 750 CFU IT) and after treatment at 24 hours with 5 mg/kg ciprofloxacin from the ciprofloxacin polymeric prodrug or free ciprofloxacin and compared to PBS vehicle controls. (B) Alveolar macrophages were quantified in the right lung and (C) bacterial burden was quantified in the left lung. Bars and error bars are presented as the median and interquartile range, respectively. Statistical significance was assessed by Kruskal-Wallis test with Dunn’s multiple comparisons. (D) Multivariate analysis comparing for individual mice left lung bacterial burdens (x-axis), % neutrophils (y-axis; % of CD45+ cells), and % alveolar macrophages (size of symbol, % of CD45+, non-neutrophils).


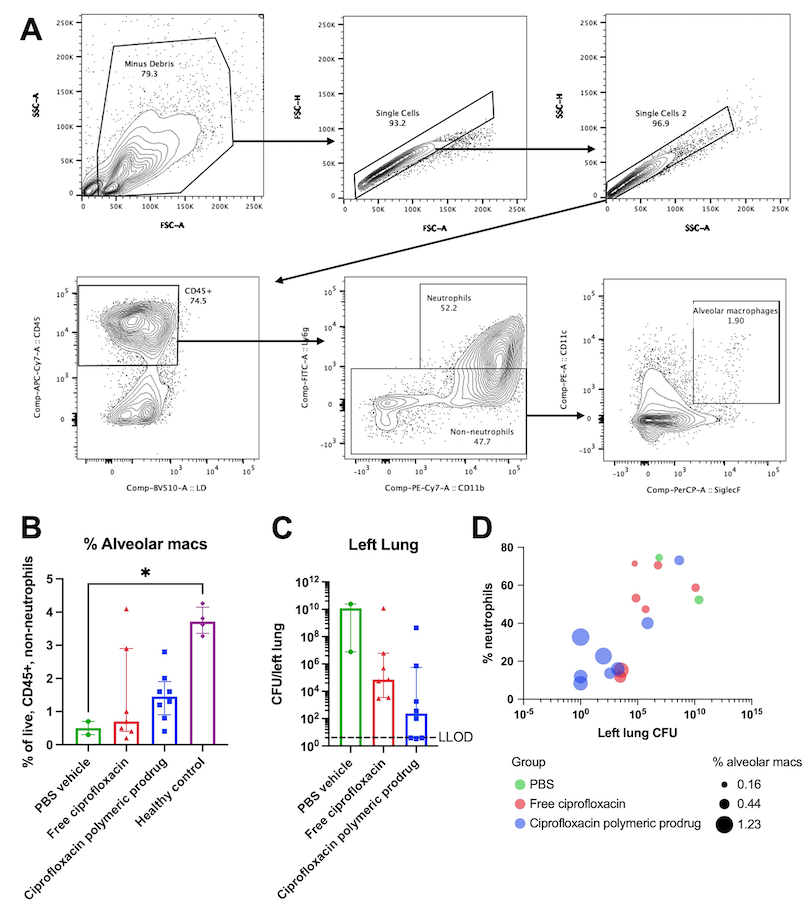


**Table S3.** Summary of target and experimental inoculum for *in vivo* experiments. Colony forming units (CFU).

**Supplemental References**

1. Misharin, A. V.; Morales-Nebreda, L.; Mutlu, G. M.; Budinger, G. R.; Perlman, H., Flow cytometric analysis of macrophages and dendritic cell subsets in the mouse lung. *Am J Respir Cell Mol Biol* 2013**,** *49* (4), 503-10.

2. Long, M. E.; Gong, K. Q.; Eddy, W. E.; Volk, J. S.; Morrell, E. D.; Mikacenic, C.; West, T. E.; Skerrett, S. J.; Charron, J.; Liles, W. C.; Manicone, A. M., MEK1 regulates pulmonary macrophage inflammatory responses and resolution of acute lung injury. *JCI Insight* 2019**,** *4* (23).
